# Tuberculosis-Associated HLH in an 8-Month-Old Infant: A Case Report and Review

**DOI:** 10.3389/fped.2020.556155

**Published:** 2020-10-30

**Authors:** Holger Hauch, Susanne Skrzypek, Wilhelm Woessmann, Kai Lehmberg, Stephan Ehl, Carsten Speckmann, Emmanuel Schneck, Dieter Koerholz, Christian Jux, Christoph Neuhäuser

**Affiliations:** ^1^Department of Pediatric Hematology and Oncology, University Children's Hospital of Giessen, Giessen, Germany; ^2^Department of Pediatric Cardiology and Intensive Care, University Children's Hospital Giessen, Giessen, Germany; ^3^Division of Pediatric Stem Cell Transplantation, University Children's Hospital Hamburg, UKE, Hamburg, Germany; ^4^Center for Chronic Immunodeficiency, Institute for Immunodeficiency, Medical Center—University of Freiburg, Freiburg, Germany; ^5^Center of Pediatrics and Adolescent Medicine, Medical Center—University of Freiburg, Freiburg, Germany; ^6^Department of Anesthesiology and Intensive Care Medicine, University Children's Hospital Giessen, Giessen, Germany

**Keywords:** tuberculosis, infant, hemophagocytic lymphohistiocytosis, lung failure, extracorporeal membrane oxygenation, ECMO, case report

## Abstract

Hemophagocytic lymphohistiocytosis (HLH) is a rare immunological disease, which can be mistaken for sepsis easily. Among the infectious causes that may trigger secondary HLH, tuberculosis (TBC), a rather rare pathogen nowadays, is typical. To our knowledge, this is the first case report of an infant suffering from TBC-associated HLH-induced acute respiratory failure who was treated successfully using extracorporeal membrane oxygenation. An 8-month-old boy with fever (over the last 8 wk) and pancytopenia was transferred to our institution with acute respiratory failure and for extracorporeal membrane oxygenation therapy. Bone marrow biopsy revealed hemophagocytosis. Immunological work-up for familial HLH was negative. In a desperate search for the cause of secondary HLH, an interferon-gamma release assay for TBC returned positive. However, microscopy for acid-fast bacteria as well as polymerase chain reaction for TBC were initially negative. Despite this, the child was treated with tuberculostatic therapy. TBC was finally confirmed. The child remained on extracorporeal membrane oxygenation for 28 d. Further work-up showed typical lesions of disseminated TBC. The mother was identified as the source of TBC. The boy presents with mild sequelae (fine motor skills). In infants with suspected septicemia, TBC should be considered as differential diagnosis even if the results are initially negative.

## Introduction

Hemophagocytic lymphohistiocytosis (HLH) is a rare syndrome, characterized by a highly stimulated but ineffective immune response. The mechanism involves an inherited or acquired defect in the handling of antigenic factors (infectious, cancerous, or autoimmune) that leads to a severe systemic inflammatory process due to T-lymphocyte proliferation, cytokine over-production, and massive macrophage activation (1, 2). The associated pancytopenia results from hypercytokinemia and hemophagocytosis that develops within the bone marrow, whereas the subsequent organ dysfunction is caused by tissue infiltration of active immune cells. The main symptoms of HLH are prolonged fever, neutro- and thrombocytopenia, hepatosplenomegaly, and conspicuous laboratory values, such as low fibrinogen and elevated levels of ferritin, triglycerides and sCD25 (3).

We report here the first case of secondary HLH in an infant with miliary tuberculosis (TBC) and acute respiratory failure, who survived following extracorporeal membrane oxygenation (ECMO) therapy. The purpose of this report is to improve recognition of this uncommon, life-threatening condition and to avoid possible pitfalls. In addition, a literature search (performed for “hemophagocytic histiocytosis,” “haemophagocytic lymphohistiocytosis,” “histiocytosis,” “hemphagocytic syndrome,” “haemophagocytic syndrome,” “histiocytic hemophagocytosis,” “histiocytic haemophagocytosis,” “children,” “infant,” “TBC,” “*Mycobacterium tuberculosis*,” “acute respiratory distress syndrome,” and “ECMO,” including databases: PubMed, EMBASE, Cochrane Library, searching period 2000–2009) provided historical and current information on this topic for our discussion on the possible pitfalls of missed diagnosis of this condition.

## Case Description

### Case History

A previously healthy 8-month-old boy presented to an outside hospital with high fever and cough since weeks. Because of C-reactive protein test showing elevated level and unspecific infiltration detected on chest x-ray, the child had been administered antibiotic therapy, consisting of beta-lactams and glycopeptides. Beside reduced levels of hemoglobin (5.6–7.8 mmol/L; normal range: 6.3–7.8 mmol/L) and platelets (90–125 × 10^9^/L; normal range: 206.000–445.000 × 10^9^/L), laboratory results were otherwise unremarkable. Results of infectious work-up [blood, cerebral spinal fluid and urine cultures, as well as polymerase chain reaction (PCR) for respiratory viruses and pathogens for atypical pneumonia] remained negative. Because of the persisting fever, the child was transferred to three other hospitals, recently to a tertiary care hospital, where he was evaluated for immunodeficiency. Bronchoscopy with bronchoalveolar lavage was carried out under general anesthesia with endotracheal intubation because of declining general condition. Since respiratory functions had deteriorated dramatically, the child was transferred to our institution for ECMO-support (at 4 wk after first presentation). No definite diagnosis existed to that time.

Medical history taking with the parents revealed an uneventful pregnancy and birth in Germany. The parents originated from the Balkan peninsula. The patient was the first child and the parents claimed to be healthy.

### Physical Examination

The child was unconscious due to deep analgo-sedation (Glasgow coma score of 3/15). The vesical temperature was 40.3°C, heart rate was 142 bpm, blood pressure was 82/44 mmHg (under continuous infusion with epinephrine 2.0 mcg/kg/min), weight was 7.9 kg (15th percentile, World Health Organization scale), length was 70 cm (30th percentile, World Health Organization scale), SpO_2_ was 70%. The pressure-controlled ventilation maintained peak inspiratory pressure of 60 cm H_2_O, positive end-expiratory pressure of 12 cm H_2_O, FiO_2_ of 1.0, frequency of 40 pm, and Horovitz quotient of 30. The spleen was 5 cm and the liver 6 cm, palpable in midclavicular lines. Our first clinical impression was that of a systemic infection with severe acute respiratory failure and unexplained lymphoproliferation.

### Laboratory Examinations

Blood analysis revealed pancytopenia (hemoglobin: 4.3 mmol/L; leucocytes: 2.600 × 10^9^/L; neutrophils: 800 × 10^9^/L; platelets: 32.000 × 10^9^/L), elevated levels of C-reactive protein (194 mg/L; normal range: <5 mg/L), ferritin (1,020 μg/L; normal range: 14–103 μg/L), triglycerides (3.58 mmol/L normal range: 0.4–1.6mmol/L), and soluble interleukin-2 receptor (30,247 U/mL; normal range: 158–623 U/mL). Analyses of arterial blood gases showed pH of 6.9, pCO_2_ of 93 mmHg, pO_2_ of 30 mmHg, and base-excess of −11.1 mmol/L.

### Imaging Studies and Further Examinations

**Figure 1:**
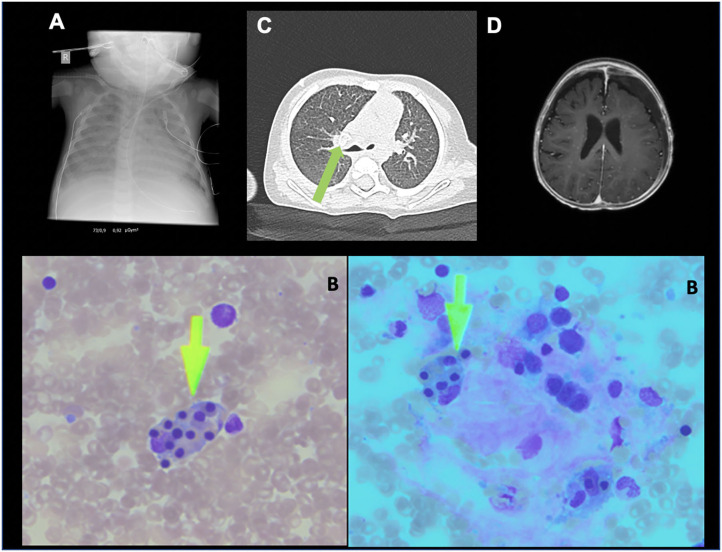
**(A)** patient's chest X-ray ad admission with acute respiratory distress syndrome, **(B)** bone arrow smear (Pappenheim stain) with hemophagocytosis (arrow), **(C)** patient's CT-scan after ECMO (day 30): calcified lymph node (arrow), **(D)** patient's MRI-scan after ECMO (day 42): small hygroma and CNS-miliary tuberculosis.

Initial imaging with x-ray showed a “white lung” (Figure 1A) and with ultrasound showed hepato- and splenomegaly. Further imaging work-up, with either computed tomography or magnetic resonance imaging, could not be performed because of the instable situation.

Bone marrow biopsy showed a distinct hemophagocytosis upon cytologic evaluation (Figure 1B). The lymphocyte population consisted of CD3^+^ of 1,358 × 10^9^/L, CD4^+^ of 1,119 × 10^9^/L, CD8^+^ of 2,017 × 10^9^/L, CD19^+^ of 487 × 10^9^/L, and CD56^+^ of 2 × 10^9^/L. Further immunological studies by fluorescence-activated cell sorting revealed normal fresh degranulation of natural killer cells (14.7%; normal: >10%), and normal expressions of perforin, CD27, SAP, and XIAP on d 7. Microscopy of the patient's hairs showed no anomaly of pigmentation, as would be found in children with Griscelli syndrome.

The patient was further evaluated with cultures of blood, urine, tracheal secretion, and stool. Examinations for ova or parasites provided negative results. All serological and PCR tests for viruses (Epstein–Barr, human immunodeficiency, cytomegaly, hanta, adeno, influenza a/b, respiratory syncytial, herpes simplex, corona, metapneumonia, parvo B19, parainfluenza, rhino, and enterovirus) were negative. Also negative were results of testing for leishmania, legionella, aspergillus, *Pneumocystis jirovecii, Hemophilus influenza* b, and pneumococcus-PCR/immunoglobulins (IgG/IgM).

All tumors markers α-fetoprotein (2.5 IU/mL), neuron-specific enolase (10 ng/mL), ß-hCG (0.2 mIU/mL), homovanillic acid (10 mg/g creatinine), and vanillylmandelic acid (18 mg/g creatinine) were unrevealing.

A positive result from the interferon-gamma release assay (IGRA) was the first indication for possible TBC infection on d 12. However, microscopy for acid-fast bacilli (Ziehl–Neelsen stain) as well as PCR testing for TBC in the first bronchoalveolar lavage were both negative on d 6. Further along in the clinical course, PCR for TBC became positive and the bronchoalveolar lavage fluid culture returned positive results on d 40. The identified strain of *M. tuberculosis* was found to be sensitive to rifampicin, ethambutol, pyrazinamide, and isoniazid, retrospectively.

After discontinuation of the ECMO therapy, further imaging was performed. Magnetic resonance imaging of the central nervous system showed a miliary TBC and a computed tomography scan of the thorax revealed a calcified, round lesion consistent with TBC (Figures 1C,D).

Normal functional assays virtually excluded hereditary forms of HLH. A secondary form was therefore recommended to be considered, as was a complete diagnostic re-evaluation (infection, tumor, leukemia). The etoposide (VP-16, according to the HLH-04 protocol) was terminated after three doses administered according the HLH protocol.

### Treatment

**Figure 2:**
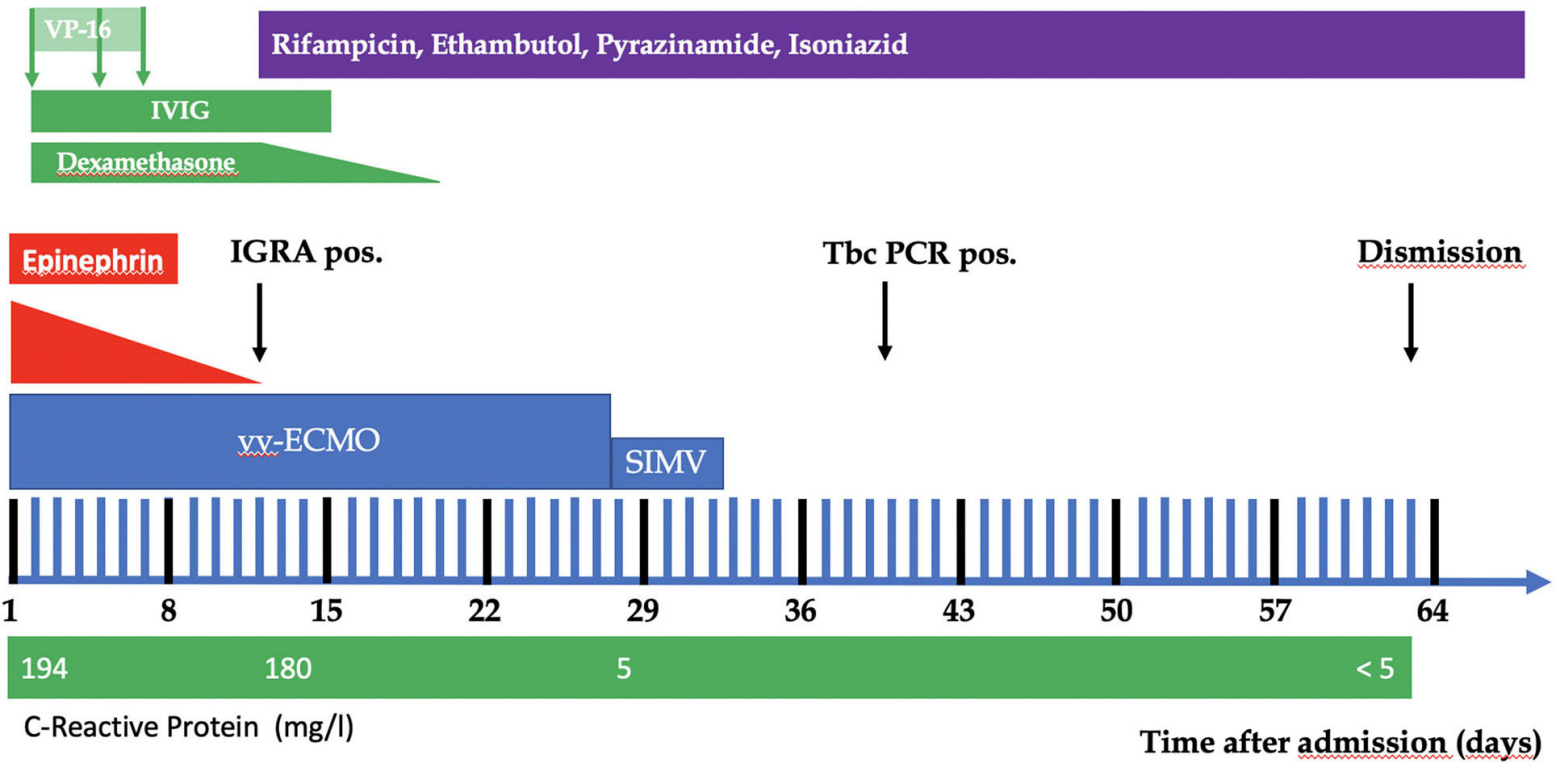
Course of the patient.

The child presented with severe hypoxia, and respiratory and metabolic acidosis, despite pressure-controlled ventilation and attempts of high frequency oscillation ventilation, both at high inspiratory oxygen level. The decision was therefore made to start veno-venous ECMO immediately after admission of the patient (see Figure 2). A 16F Avalon double-lumen cannula (Getinge AB, Gothenburg, Sweden) was inserted in the right internal jugular vein. A blood flow of 120 mL/kg/min was established, which was sufficient to achieve a PaO_2_ between 85 and 95 mmHg, and a PaCO_2_ between 40 and 50 mmHg. Ventilator parameters were reduced as much as possible (peak inspiratory pressure of 16 cm H_2_O, positive end-expiratory pressure of 6 cm H_2_O, and FiO_2_ of 0.4) to guarantee “lung protection” as well as a transcutaneous O_2_-saturation of >90%. After initiation of the ECMO catecholamine support with epinephrine and noradrenaline (maximum dosage of 0.18 μg/kg/min) was progressively weaned (until d 12).

Because of the fulfillment of criteria for HLH therapy, the HLH-94 protocol was initiated. The boy was treated with dexamethasone at 10 mg/m^2^ (d 2 to d 12, and reduced/discontinued at d 24) and etoposide at 150 mg/m^2^ (three doses in total, given on d 2 through d 7 and being stopped upon normal results from the T cell degranulation assay on d 7). Additionally, intravenous immunoglobulin (0.5 g/kg body weight, given on d 2 through d 15) was administered.

With the suspicion of TBC, on d 12 (positive IRGA) a 4-fold tuberculostatic therapy was started, consisting of rifampicin at 1 × 15 mg/kg body weight/d, ethambutol at 1 × 30 mg/kg body weight/d, pyrazinamide at 1 × 30 mg/kg body weight/d, and isoniazid 1 × 10 mg/kg body weight/d). At 10 d after the start of tuberculostatic therapy, there was improvement in the general condition (stable cardiorespiratory situation, rising tidal volumes). The C-reactive protein level declined from 194 mg/L to 5 mg/L on d 28, followed by the soluble interleukin-2 receptor from 30,247 U/mL to 931 U/mL on d 40. After full lung recovery, the ECMO support was able to be discontinued on d 28. The child was extubated on d 33.

### Outcome and Follow-Up

The boy survived this critical condition. After discharge on d 66, the child was re-evaluated on d 98, d 460, and d 745. During that time, the boy developed frontotemporal hygroma (left side > right side) with spontaneous resolution. Neurological assessment during the follow-up period revealed a mild retardation of development (speech and fine motor skills) and moderate aggressive behavior with beginning improvement.

## Discussion

This case report shows a rare combination of two so-called “mimickers.” TBC is known as a “great mimicker” (4) but HLH can also be mistaken as sepsis (5–7). The HLH syndrome is characterized by an inadequate immune response, with proliferation and activation of macrophages that leads to an exorbitant phagocytosis of hematologic precursors and erythrocytes, platelets, etc. (8). It can occur as a primary disorder (F-HLH) caused by different genetic mutations [i.e., PRF, UNC13D, STXBP2, STX11, and RAB27A (9)], or as a secondary sporadic disorder triggered by infection, autoimmune diseases, pregnancy, or malignant diseases (Table 1) (10–37, 46–49). In our case, the young age and the massive involvement of the lung, the high activity of the disease (high laboratory values for ferritin and the surface interleukin-2 receptor) led us to the suspicion of primary, hereditary HLH. The cytotoxic drug etoposide (VP-16, a semisynthetic derivative of podophyllotoxin) was therefore started, according the HLH-94 protocol, after informed consent was obtained from the parents (50). In the same period, degranulation assays were carried out to verify the suspicion of hereditary HLH. Familial forms of HLH are identifiable by functional immunological tests (natural killer cell and cytotoxic T lymphocyte degranulation, and expression of perforin, SAP, and XIAP), allowing a rapid and reliable classification of affected patients (51). After exclusion of hereditary HLH on d 7, the VP-16 was stopped to avoid its side effects, such as bone marrow suppression or secondary infection. It was very important for us to diagnose or exclude f-HLH because of the cytostatic treatment. Not only were expression of perforin (FHL2), CD27, SAP (XLP1), and XIAP (XLP2) determined to be normal. Also NK cell degranulation assays were performed with normal results to exclude further cytotoxicity defects which do include Munc13-4 (FHL3), STX11 (FHL4), Munc18-2 (FHL5), RAB27A (GS2), LYST (CHS). These fully normal test virtually exclude these deficiencies, which is why—following a well-established diagnostic algorithm—genetic testing was not required.

**Table 1:** Causes of sporadic forms of HLH.

**Infectious agents**				**Malignancy**	**Others**
**Viral**	**Bacterial**	**Fungal**	**Parasitic**		
Adenovirus (10)	*Hemophilus* *influenza* (11)	*Candida albicans* (12)	Leishmania (13)	Non-Hodgkin lymphoma (14)	MDS (15)
Cytomegalovirus (16)	*Mycoplasma* *pneumoniae* (17)	*Cryptococcus neoformans* (18)	*Plasmodium* spp. (19)	Acute lymphoblastic leukemia (20)	Pregnancy (21)
Epstein–Barr virus (22)	*Staphylococcus* spp. (23)	*Histoplasma capsulatum* (24)		Hodgkin's disease (25)	Rheumatic diseases (26, 27)
Enterovirus (28)	*Streptococcus* spp. (29)	*Fusarium oxysporum* (30)		Acute myeloid leukemia (31)	
Herpes simplex virus (32)	*Brucella* spp. (33)			Chronic lymphatic leukemia (34)	
Human immunodeficiency virus (35)	*Coxiella* spp. (36)				
Influenza virus (37)	*Mycobacterium tuberculosis* (38–45)				
Parvovirus B19 (46)	Mycobacteria other than TBC, MOTT (47)				
Varicella virus (48)					
Measles virus (49)					

*HLH, hemophagocytic lymphohistiocytosis, MDS, myelodysplastic syndrome; MOTT, mycobacteria other than tuberculosis*.

Reports in the literature argue the pros and cons of chemotherapy for treatment of secondary HLH. Fatal outcomes after usage of chemotherapy have been reported for cases of Epstein–Barr virus-associated HLH (52). On the other hand, one report showed chemotherapy to be beneficial, even with pregnancy-associated HLH (53). Thus, the use of VP-16 remains a case-by-case decision, based upon weighing up the severity of the disease with the possible side effects.

The limitation of this report is that TBC is known to trigger secondary HLH. We found 8 other cases with TBC and HLH in neonates and infants (38–45) (Table 2) but none of them need ECMO-support. Most of these patients presented with respiratory failure. Half of them received a specific treatment for HLH (e.g., immunoglobulins, dexamethasone). In 7 of the 8 cases, TBC was diagnosed in a timely manner, and the appropriate tuberculostatic therapy was started. From these reports, however, survival seemed to be more related to the TBC treatment than to the HLH treatment. In our case, the ECMO support guaranteed immediate survival, buying time to identify the underlying disease, to initiate a specific treatment, and to promote healing. Without these (pre-)conditions, ECMO therapy has no purpose (*per se*) and is very probably futile. We conclude, that even though diagnosis and treatment of TBC-associated HLH is difficult and outcome is uncertain, ECMO support should not be withheld from these very sick patients. Disorders of Mendelian Susceptibility to Mycobacterial Disease predominantly predispose to non-tuberculous mycobacteria and were thus not further investigated. The fact that the patient has remained well and asymptomatic for >4 years, underscores that HLH was of secondary nature and no predisposition syndrome is present.

**Table 2:** Published cases of TBC-induced HLH in neonatal age or infancy.

**Reference/year**	**Birth country**	**Origin**	**Sex**	**Age of onset**	**Respiratory failure**	**Site of TBC isolation**	**Detection method for MTb**	**HLH therapy**	**TBC therapy**	**Survival**
**NEONATES**										
(38)/2001	United States	Nigeria	F	12 d	Yes (MV)	BAL, puncture (liver)	Microscopy pos. for AFB	No	Yes (INH, Rifa, Etam, Pyra)	Yes
(39)/2003	Thailand	Thailand	M	2 wk	Yes (MV)	BAL, gastric aspiration	Microscopy pos. for AFB	No	Yes (Strep, Oflox, INH, Etam, Rifa)	Yes
(40)/2012	India	India	F	3 wk	Yes (MV)	BAL	Microscopy pos. for AFB, PCR pos. for MTb	Yes (Dexa only)	Yes (INH, Rifa, Pyra)	Yes
(41)/2016^*^	Australia	India	M	3 wk	Yes (MV)	BAL^§^	Microscopy neg. for AFB, PCR pos. for MTb, IRGA neg. Culture pos.	Yes (Dexa only)	Yes (INH, Rifa, Oflox, Pyra)	Yes
**INFANTS**										
(42)/2000	United States	Latin America	F	7 wk	Yes (MV)	BAL^§^	Microscopy neg. Culture pos.	No	No	No
(43)/2008	India	India	M	7 wk	Yes (MV)	Gastric aspiration	Microscopy pos. for AFB	Yes (IVIG only)	Yes (INH, Rifa, Etam, Pyra)	No
(44)/2010	India	India	M	2 mo	No	Bone marrow aspiration	Microscopy pos. for AFB	No	Yes (INH, Rifa, Etam, Pyra)	Yes
(45)/2014	India	India	M	2 mo	Yes (MV)	BAL	Microscopy pos. for AFB	Yes (Dexa, CSA)	Yes (Strep, Oflox, Etam)	No
Case in this article/2016^#^	Germany	Southeast Europe	M	8 mo	Yes (vv-ECMO)	BAL^§^	Microscopy neg., Second PCR pos. for MTb, IRGA pos. Culture pos.	Yes (IVIG, Dexa, VP-16)	Yes (INH, Rifa, Pyra, Etam)	Yes

*Published cases of TBC-associated HLH in infancy to date. A total of 9 cases in children have been reported (including our case). In 8/9 cases, tuberculostatic therapy was administered; in 5/9 cases, HLH-therapy was administrated. At total of 3 children died, 2 of whom were treated with HLH therapy and tuberculostatic therapy*.

# *Year of appearance*.

* *Proven congenital TBC*.

§ *TBC culture after 5-6 wk*.

*AFB, acid-fast bacilli; CSA, cyclosporin A; Dexa, dexamethasone; ECMO, extracorporeal membrane oxygenation; Etam, ethambutol; HLH, hemophagocytic lymphohistiocytosis, INH, isoniazid; IVIG, intravenous immunoglobulins; MV, mechanical ventilation Neg., negative; Oflox, ofloxacin; PCR, polymerase chain reaction; Pos., positive; Pyra, pyrazinamide; Rifa, rifampicin; Strep, streptomycin; TBC, tuberculosis; VP-16. etoposide*.

Diagnosing TBC in neonates, infants, and young children remains problematic, since false negative results are common. In our case, microscopy for acid-fast bacilli as well as the initial PCR for TBC were both negative. In contrast, the IRGA-test surprisingly returned positive. Because of the published limitations of the IRGA in children <5-years old (54), the intracutaneous skin test is still usually recommended. In most of the 8 reported cases, the TBC was diagnosed by microscopy or PCR. However, recent data suggest that the IRGA may have a role in diagnosing TBC in infants as well (55). In our case, tuberculostatic therapy was initiated on suspicion. The definite diagnosis of TBC was achieved by positive cultures, with a duration of 5 wk. Later, there was further radiological evidence of miliary lesions in the lungs, central nervous system, and lymph nodes.

## Conclusion

Few cases of TBC-associated HLH in neonates and infants are reported in the literature. Due to the high incidence of TBC in India, most of these reports originate from that country (56). However, even in high-income countries, physicians should be aware of the risk and the symptoms of TBC, which may present as severe illness in infancy, with signs of HLH and acute respiratory failure. Treatment for TBC may be started on suspicion to avoid loss of time. HLH should also be addressed specifically. In the case of refractory hypoxemia, application of ECMO meanwhile can support and bridge the patient until the specific therapy can be effective.

## Data Availability Statement

All datasets generated for this study are included in the article/supplementary material.

## Ethics Statement

Written informed consent was obtained from the minor(s)' legal guardian/next of kin for the publication of any potentially identifiable images or data included in this article.

## Informed Consent Statement

Informed written consent was obtained from the parents of the patient for publication of this report and any accompanying anonymized images.

## Care Checklist Statement

The authors have read the CARE Checklist (2013), and the manuscript was prepared and revised according to the CARE Checklist (2013).

## Author Contributions

HH was the patient's physician, reviewed the literature, and drafted the manuscript. SS, WW, ES, DK, and CN were the patient's physicians, reviewed the literature and contributed to the manuscript's drafting. CJ reviewed the literature and contributed to the manuscript's drafting. KL, SE, and CS performed the immunological analyses, and both were responsible for the revision of the manuscript for important intellectual content. All authors issued final approval for the version to be submitted.

## Conflict of Interest

The authors declare that the research was conducted in the absence of any commercial or financial relationships that could be construed as a potential conflict of interest.
